# An FPGA-Based Ultra-High-Speed Object Detection Algorithm with Multi-Frame Information Fusion

**DOI:** 10.3390/s19173707

**Published:** 2019-08-26

**Authors:** Xianlei Long, Shenhua Hu, Yiming Hu, Qingyi Gu, Idaku Ishii

**Affiliations:** 1 The Research Center of Precision Sensing and Control, Institute of Automation, Chinese Academy of Sciences, Beijing 100190, China; 2 The School of Artificial Intelligence, University of Chinese Academy of Sciences, Beijing 101408, China; 3 The Robotics Laboratory, Department of System Cybernetics, Hiroshima University, Hiroshima 739-8527, Japan

**Keywords:** ultra-high-speed vision, object detection, field-programmable gate array, histogram of oriented gradient, multi-frame information fusion model

## Abstract

An ultra-high-speed algorithm based on Histogram of Oriented Gradient (HOG) and Support Vector Machine (SVM) for hardware implementation at 10,000 frames per second (FPS) under complex backgrounds is proposed for object detection. The algorithm is implemented on the field-programmable gate array (FPGA) in the high-speed-vision platform, in which 64 pixels are input per clock cycle. The high pixel parallelism of the vision platform limits its performance, as it is difficult to reduce the strides between detection windows below 16 pixels, thus introduce non-negligible deviation of object detection. In addition, limited by the transmission bandwidth, only one frame in every four frames can be transmitted to PC for post-processing, that is, 75% image information is wasted. To overcome the mentioned problem, a multi-frame information fusion model is proposed in this paper. Image data and synchronization signals are first regenerated according to image frame numbers. The maximum HOG feature value and corresponding coordinates of each frame are stored in the bottom of the image with that of adjacent frames’. The compensated ones will be obtained through information fusion with the confidence of continuous frames. Several experiments are conducted to demonstrate the performance of the proposed algorithm. As the evaluation result shows, the deviation is reduced with our proposed method compared with the existing one.

## 1. Introduction

In fields such as robotics, remote sensing and autonomous vehicles driving, real-time object detection is the basis for target tracking. To improve the accuracy and speed of object detection, various algorithms have been proposed over the last few decades [1,2,3]. Conventional object detection relies on robust image feature descriptors, such as Binary Robust Independent Elementary Features (BRIEF) [4], Oriented FAST and Rotated BRIEF (ORB) [5] and Histogram of Oriented Gradient (HOG) [6]. The rapid development of Convolutional Neural Networks (CNNs) makes a contribution to CNN-based object detection, including Regions with CNN features (R-CNN) [7], You Only Look Once (YOLO) [8,9,10], which achieve accurate object detection and satisfactory performance in specific scenarios. Although the accuracy of CNNs is generally higher than that of methods based on features, a massive amount of labeled samples are needed for network training, which limited their application where few samples are available. In addition, the computation complexity of CNNs hinders their implementation in embedded devices.

Constrained by computational power and algorithm complexity, the processing speed of both conventional descriptor- and CNN-based algorithms is limited to dozens of frames per second, being close to the processing speed of human eyes and similar to the rate of traditional video signal format (e.g., National Television System Committee (NTSC) at 30 fps and Phase Alternating Line (PAL) at 25 fps). However, ultra-high-speed object detection is important in many fields. For example, in bio-engineering, microorganisms move fast under microscopes and are hard for humans to observe.

To increase the processing frame-rate, plenty of ultra-high-speed vision systems based on FPGA processors have been developed [11]. Likewise, many hardware oriented fast object detection algorithms have been proposed upon these platforms [12,13,14,15,16,17,18,19]. Limited on-chip memory is the major restriction to implement algorithms with highly parallel pipelines, whereas interactions with off-chip memories can substantially reduce the processing frame rate. Existing ultra-high-speed object detection methods always use basic image features and have poor robustness against illumination, scale and direction, thus limiting their application in real scenes. Hence, more effective and efficient object detection algorithms are still required for improved accuracy in complex scenarios.

The HOG descriptor [6] proposed by Dalal and Triggs was first used for pedestrian detection under complex backgrounds, achieving nearly perfect results. The HOG descriptor with support vector machine (SVM) [20] classification is widely used in various fields for robust object detection and recognition. Given its high computational complexity and resource requirements, the HOG descriptor is mainly deployed in Central Processing Units (CPUs) and Graphics Processing Units (GPUs). The transmission speed from camera to personal computers (PCs) and the computational power of modern processors impedes processing speeds above 100 fps. FPGAs are required for higher image processing speed.

Li et al. implemented an optimized HOG descriptor in high-speed vision platform SA-X2, achieving object detection at 12,000 fps. However, the stride of adjacent detection windows is 16 pixels limited by the high input pixel-parallelism. In order to reduce the location deviation caused by the large stride, a multi-frame information fusion method is proposed in this paper. The starting points presenting the detection area are adjustable among adjacent frames. Through confidence-based localization information fusion, more accurate coordinate values are obtained.

## 2. Related Work

Object detection is a fundamental task in computer vision, being strongly demanded and widely used in robotics, industrial automation, manufacturing and other fields. Several algorithms have been proposed in recent years to improve the speed and accuracy of object detection for wider applicability. These methods are generally based on either handcrafted features or CNNs, and most of them achieve satisfactory performance in different scenarios.

The SIFT descriptor [21] proposed by Lowe in 2004 is invariant to image scaling and object orientation. Hence, this descriptor is widely used in image stitching. Bat et al. proposed the Speeded Up Robust Features (SURF) to improve the performance of SIFT in terms of repeatability, distinctiveness, and robustness. As mentioned above, HOG [6] is another type of descriptor for object detection and, as well as the Binary Robust Independent Elementary Features (BRIEF) [4] and Oriented FAST and Rotated BRIEF (ORB) [5], is widely used in image stitching and object detection given its high performance.

Since AlexNet [22] won the Image Large Scale Visual Recognition Competition (ILSVRC) in 2012, many CNN architectures for object detection have been proposed [7,8,9,10,23,24,25]. These networks can be divided into two-stage and end-to-end methods, which contain a large number of parameters and achieve high accuracy in open source datasets. For instance, R-CNN [7] is a two-stage object detection method that bounding boxes are first proposed with the Selective Search (SS) [26] algorithm. In this method, a 4096-dimensional vector is obtained for classification with a CNN, and then a trained SVM performs category discrimination. Although effective, this method is time-consuming, as approximately 6–7 s are required for object detection on a single frame. Similar two-stage algorithms, such as Fast R-CNN [23], have improved the calculation speed but still remains at low processing rates. In contrast, one-stage detection algorithms such as YOLO [8,9,10] and Single Shot MultiBox Detector (SSD) [24] have substantially reduced the computation time, achieving processing rate above 50 fps. The accuracy of CNN based object detection is much higher than that with handcrafted descriptors, and intense research efforts have been devoted to the implementation of CNNs on FPGAs [27,28]. However, the large number of CNN parameters are unable to be stored in on-chip memories, and frequently accessing off-chip memory will reduce the processing speed, thus limiting the application of FPGAs for ultra-high-speed object detection.

The computational complexity of algorithms and calculation power of the processors limited the processing rate below 100 fps. However, ultra-high-speed object detection at more than 1000 fps is required for many applications (e.g., microbial tracking, cell sorting) [29]. To achieve such processing rates, many object detection algorithms have been proposed based on high-speed vision platforms. For instance, color histogram features is used in the real-time tracking system [30] proposed by Ishii et al., achieving a processing rate of 2000 fps for images of 512 × 512 pixels. Still, the system only performed well under evident contrast between foreground and background. Gu et al. proposed a multiple-object detection algorithm based on brightness information and implemented in FPGAs. The algorithm was successfully applied to a cell analysis system comprising a Lab-on-a-Chip (LOC), in which morphological information is acquired for advanced analyses. However, the feature of brightness undermined the robustness and applicability of this algorithm. To improve robustness, Li et al. proposed a HOG-based algorithm [15,16] that achieves ultra-high-speed object detection at more than 10,000 fps. Moreover, multiple objects can be detected under complex backgrounds. However, the detection windows were fixed at 48 × 48 pixels with stride of 16 pixels, and only objects located around the center of detection windows were detected. Based on the existing algorithm, the multi-frame information fusion method is introduced in this paper, with which deviation of object localization is reduced.

## 3. Ultra-High-Speed Object Detection Algorithms

### 3.1. Existing Hardware Oriented HOG Algorithm

**Figure 1:**
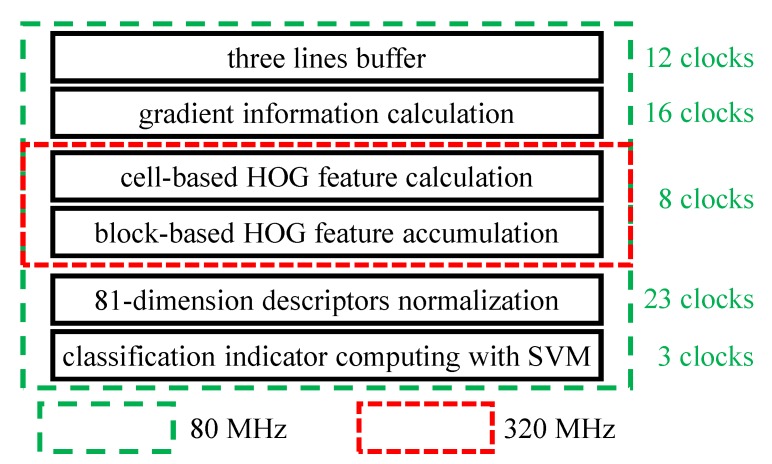
The entire processing procedure of the previous algorithm.

The HOG descriptor [6] retrieves nearly perfect accuracy for pedestrian detection. This descriptor basically represents objects in images by the distribution of intensity gradients and edge directions. However, the expensive computation impedes to obtain HOG descriptors at rates above 100 fps in CPU or GPU implementations. To achieve ultra-high-speed object detection under complex backgrounds, Li et al. [15,16] optimized the HOG descriptor and deployed it in the high-speed vision platform SA-X2. The algorithm achieves processing rates of 10,000 fps through some optimization, and its procedure is shown in Figure 1.

First, gradient magnitude (g(x,y)) and orientation (θ(x,y)) of each pixel (I(x,y)) are calculated as follows:

(1) $$\begin{array}{c} \left\{\begin{array}{c} f_{x}\left(x,y\right)=I\left(x+1,y\right)-I\left(x-1,y\right)\\ f_{y}\left(x,y\right)=I\left(x,y+1\right)-I\left(x,y-1\right), \end{array}\right. \end{array}$$

(2) $$\begin{array}{c} g\left(x,y\right)=\sqrt{f_{x}^{2}\left(x,y\right)+f_{y}^{2}\left(x,y\right)}, \end{array}$$

(3) $$\begin{array}{c} \theta \left(x,y\right)=\arctan\left(\frac{f_{y}\left(x,y\right)}{f_{x}\left(x,y\right)}\right), \end{array}$$

where I(x±1,y±1) is the grayscale value of the surrounding pixels.

**Figure 2:**
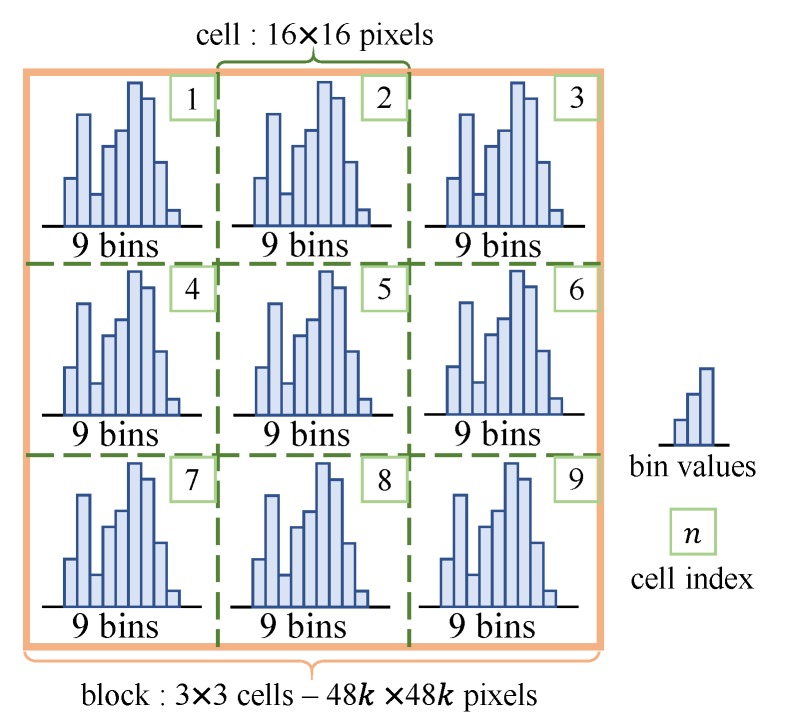
Architecture of blocks and cells in the existing algorithm.

Then, the gradient magnitude of each pixel is divided into nine bins according to its orientation at intervals of 20${}^{\circ }$, and the magnitude is accumulated for pixels in the same cell, forming a nine-dimensional histogram ($C_{hog}$). As shown in Figure 2, the cells are defined as regions of 16 × 16 pixels, and both the detection window and block are composed of 3 × 3 cells.

Next, an 81-dimensional descriptor ($B_{hog}$) is obtained through concatenating the original HOG descriptors in the same blocks as follows:

(4) $$B_{hog}=v_{1}^{\prime }\cup v_{2}^{\prime }\cup ...\cup v_{n-1}^{\prime }\cup v_{n}^{\prime },$$

where *n* is the cell index in a given block.

For improved accuracy and guaranteeing invariance to factors such as illumination and shadowing, normalization is conducted within blocks. By considering both accuracy and computational cost, the L2 normalization method described in Equation (5) is adopted for hardware implementation:

(5) $$v^{\prime }=\frac{v}{\sqrt{{∥v∥}_{2}^{2}+\epsilon }},$$

where *v* is the original HOG feature of a cell in given block and $v^{\prime }$ is the normalized one, whereas ϵ prevents division by zero. After normalization, an integrated linear SVM judges whether the target object is present in the detection window by comparison with a threshold. Several experiments have demonstrated the high performance of the optimized HOG descriptor in detection of textured objects over complex backgrounds.

However, the pixel parallelism is 64 pixels, and the stride between detection windows is 16 pixels, limited by the high pixel parallelism, which is slightly large considering object sizes of approximately 48 × 48 pixels. The evaluations and experiments reported in [16] show that objects located in the center of the detection windows are easily recognized, whereas those located off-center retrieve unreliable detection. Therefore, we propose an improvement to this algorithm as detailed in the sequel.

### 3.2. Proposed HOG Descriptor

To address the large stride between detection windows from [16], we propose the detection area spanning method. The proposed algorithm is based on the existing hardware oriented HOG descriptor, but the starting point of detection area is adjustable, aiming to decrease the stride between detection windows. Unlike the existing algorithm, we do not store all the HOG features of a frame but only the largest one and the corresponding coordinates of the consecutive frame in the bottom of the images.

To this end, a signal and data regeneration module is added before the hardware oriented algorithm to provide a synchronization signal indicating the detection area. In addition, to keep alignment with the synchronous signal, image data are regenerated by concatenation with those from the preceding clock cycle.

The existing hardware oriented HOG algorithm has fixed starting point ($x_{s},y_{s}$) for the detection window, whereas the stride between detection windows is given by *S*, and the τ-th frame is denoted as O(τ). The proposed algorithm considers a varying detection area among frames with starting point being calculated as Equation (6):

(6) $$\left(x_{s}^{\prime },y_{s}^{\prime }\right)=\left(x_{s}+\left(\left\lfloor \tau /n\right\rfloor \%n\right)\times \frac{S}{n},y_{s}+\left(\tau \%m\right)\times \frac{S}{m}\right),$$

where ($x_{s},y_{s}$) is the starting point of the detection area from the existing algorithm, ($x_{s}^{\prime },y_{s}^{\prime }$) is that from the proposed algorithm in frame O(τ), and ⌊x⌋ is the drop-down integer operation upon *x*. The cell size is divided into several parts along the horizontal and vertical directions with parameters *m* and *n* satisfying Equation (7):

(7) $$\begin{array}{cc} 0<n<S & n\%S=0,\\ 0<m<S & m\%S=0. \end{array}$$

**Figure 3:**
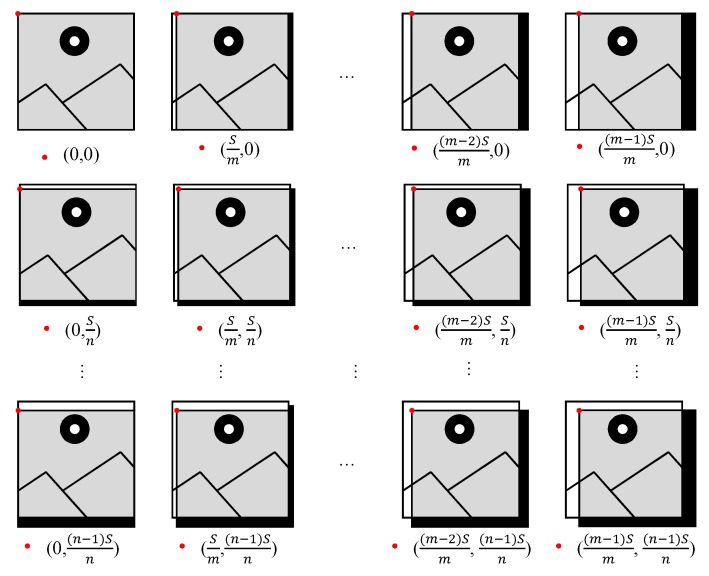
Detection area variation among adjacent frames.

In the existing hardware oriented algorithm, the starting point is (0,0), and the stride among detection windows *S* is fixed to 16 pixels. In the proposed variant of the algorithm, starting point ($x_{s}^{\prime },y_{s}^{\prime }$) of detection window is cycled among (0,0), ($\frac{S}{m}$,0), ⋯, ($\frac{(m-1)S}{m}$,0), (0,$\frac{S}{n}$), ($\frac{S}{m}$,$\frac{S}{n}$), ⋯, ($\frac{(m-1)S}{m}$,$\frac{S}{n}$), ⋯, (0,$\frac{(n-1)S}{n}$), ($\frac{S}{m}$,$\frac{(n-1)S}{n}$), ⋯, ($\frac{(m-1)S}{m}$,$\frac{(n-1)S}{n}$), as described in Figure 3. The starting points different from (0,0) make a small portion of the detection area to remain uncovered and introduce small invalid areas. For starting points (*x*, *y*), totally xN+yM−xy pixels are uncovered, occupying $\frac{xN+yM-xy}{MN}$ of the entire image frame, here *M* and *N* are the size of the image. In fact, the proportion of uncovered area is the same as that of an invalid area. Considering the feature that only one frame is transmitted to a PC for every four frames, *m* and *n* are set to 2 in our hardware implementation, and the starting points are cycled among (0,0), (8,0), (0,8) and (8,8). For starting points (8,0) and (0,8), about 1.56% of an image is uncovered and the percent is 3.1% for starting point (8,8) when the image size is 512 × 512 pixels. These areas still have a negligible impact on object detection.

**Figure 4:**
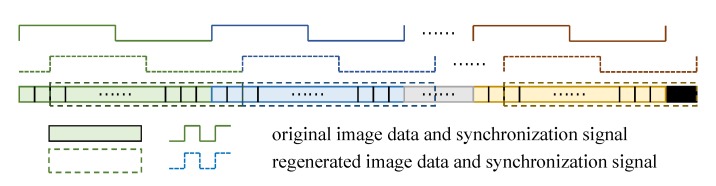
Original image data, synchronization signal and the regenerated ones.

Figure 4 depicts the image data regeneration module. The original data and synchronization signal are represented by solid lines, and the regenerated data are shown in dotted lines. Image data, whether original or generated, are always synchronized with the clock signal. Zero padding is performed for the pixels exceeding the boundaries of the captured image.

**Figure 5:**
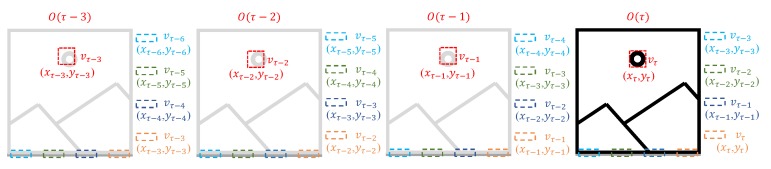
HOG feature storage in adjacent frames for the proposed algorithm.

The remaining steps for the HOG feature calculation procedure are described in Figure 1. First, including the surrounding image data, 198 pixels are buffered into the first three lines of a buffer module. The gradient magnitude and orientation of each pixel are calculated on the buffered pixels. The magnitude of each pixel is categorized into different bins according to its orientation. Then, gradient magnitudes in the cells with the same orientation bin are accumulated, forming a nine-dimensional histogram that indicates the distribution of gradient direction within pixels. Next, the histograms of each cell in a given block are concatenated, forming an 81-dimensional, vector. Meanwhile, the squares of each HOG feature value within the block are accumulated. Normalization is conducted upon the 81-dimensional, vector using the sum of squares. Finally, multiplication with integer SVM parameters generates a 32-bit signed value representing the existence of the target object within the detection window. In the existing hardware oriented HOG algorithm, all of the HOG features are stored in the bottom of the image and a small part of image is covered to fulfill the storage requirements. Then, further processing is performed on a PC using the recorded HOG feature values. Given the limited transmission bandwidth, only a quarter of the images can be transmitted to the PC, losing the information from three out of four frames. To prevent this type of information loss, we adopt another post-processing strategy by only storing the maximum HOG feature value and corresponding coordinates per frame. The values are stored in the bottom of the image along with those from the previous three frames, as illustrated in Figure 5. Frame O(τ) (shown in color of black) can be processed and then transmitted to a PC, whereas other frames are processed in the FPGA. Here, $v_{\tau }$ represents the maximum HOG feature value within frames and ($x_{\tau }$, $y_{\tau }$) are the corresponding coordinates.

The information from the previous three frames is utilized for location compensation with confidence of HOG feature values ($p_{n}^{v}$) and confidence of frames ($p_{n}^{f}$), aiming to improve the detection accuracy. The confidence of HOG feature values is defined as Equation (8):

(8) $$p_{n}^{v}=v_{n}/v_{max},$$

where $v_{max}$ is the maximum value among $v_{\tau }$, $v_{\tau -1}$, $v_{\tau -2}$ and $v_{\tau -3}$. The confidence of frames is defined as in Equation (9):

(9) $$p_{n}^{f}=1-\left(\tau -n\right)/4\times 100\%.$$

Clearly, the current frame has the highest confidence. The same as in Equation (8), *n* is the index of image frame. Then, the confidence of each coordinate is defined as in Equation (10):

(10) $$p_{n}=p_{n}^{v}\times p_{n}^{f}.$$

The compensated coordinate of the detected object is calculated with Equation (11):

(11) $$\left(x_{c},y_{c}\right)=\left(\frac{\sum_{\tau -3}^{\tau }p_{n}\times x_{n}}{\sum_{\tau -3}^{\tau }p_{n}},\frac{\sum_{\tau -3}^{\tau }p_{n}\times y_{n}}{\sum_{\tau -3}^{\tau }p_{n}}\right).$$

When the target object for detection moves fast, the previous detected locations will undermine the detection performance. Hence, we introduced fusion method when the maximum distance below threshold θ among the coordinates of current and the previous three frames:

(12) $$\left(x_{o},y_{o}\right)=\left\{\begin{array}{cc} \left(x_{\tau },y_{\tau }\right) & d_{m}\leq \theta ,\\ \left(x_{c},y_{c}\right) & d_{m}>\theta , \end{array}\right.$$

where $d_{m}$ is the maximum distance of the four detected object locations, ($x_{\tau },y_{\tau }$) is the detected location of object in the current frame, and ($x_{c},y_{c}$) is the compensated location.

## 4. Implementation

### 4.1. Hardware Platform

The hardware platform used for algorithm implementation is a high-speed camera FASTCAM SA-X2, which is the product of Photron Ltd., Tokyo, Japan, aiming at ultra-high speed image recording. The maximum resolution is 1024 × 1024 pixels, and the maximum frame rate is up to 10,000 fps under full resolution. The input parallelism is 64 pixels with each pixel characterized by 12 bits and the entire data throughput is up to 117.19 Gbits. The image data collect from Complementary Metal Oxide Semiconductor (CMOS) sensor are stored in embedded Double Data Rate Synchronous Dynamic Random Access Memories (DDRs). In order to process image data and transmit processed results to a PC in real-time, an external board embedded with an Altera Stratix IV FPGA (San Jose, CA, USA) is developed. The image size in the external board is down-sampled to 512 × 512 pixels limited by the transmission bandwidth from the camera body to the external board. With four CoaxPress protocol cables, a part of images can be transmitted to PC for post-processing. Given the limited transmission speed, images of 512 × 512 pixels (256 × 256 pixels) can be transmitted at 2500 fps (10,000 fps) from the external board to a PC. To better evaluate the proposed algorithm, images of 512 × 512 pixels are used for object detection.

The PC is also used for SVM parameter training and to conduct evaluations and experiments. The PC is an ASUS P9X79-E WS mainboard with an Intel Core i7-4930K 3.40 GHz CPU (Santa Clara, CA, USA) and 16 GB memory, running the Microsoft Windows 7 Ultimate 64-bit operating system (Redmond, DC, USA).

### 4.2. Hardware Implementation

**Figure 6:**
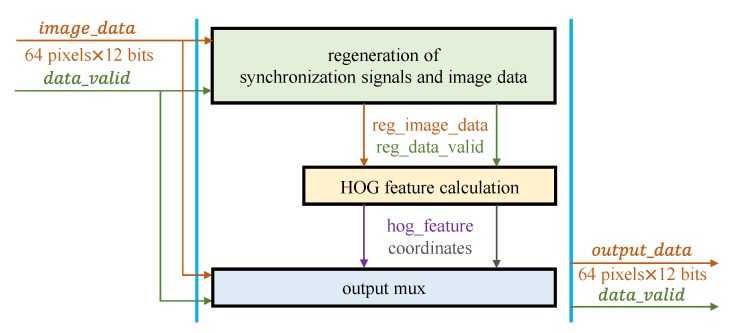
Image processing architecture for the proposed object detection algorithm.

Image processing of the proposed algorithm can be divided into three sub-modules: regeneration of synchronization signals and image data, HOG feature calculation and output multiplexing. The input of the image processing module is the image data (64 pixels ×12 bits) and the synchronization signal, and the output is either image data or HOG features. The proposed image processing architecture is illustrated in Figure 6.

Establishing the synchronization signal and regenerating image data represents the first step in image processing and aims to redefine the detection area.

**Figure 7:**
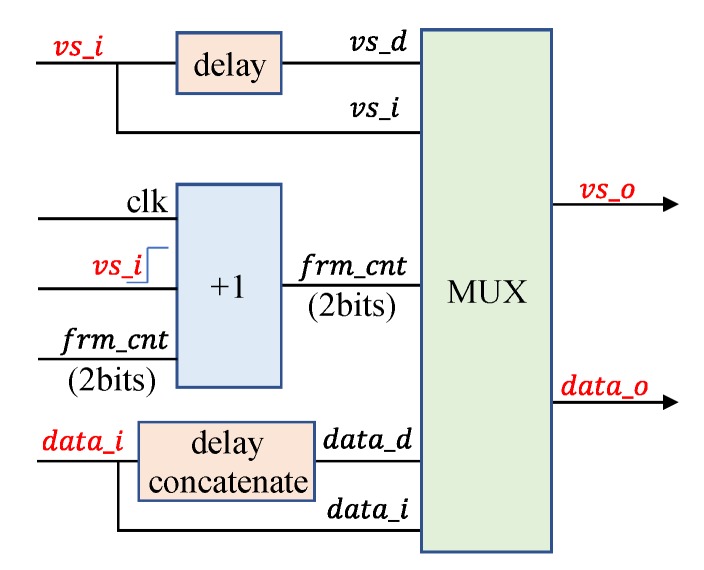
Signal and data regeneration module.

As shown in Figure 7, vs_d and data_d can be obtained from vs_i and data_i through time-delay circuits, whereas frm_cnt, which is represented by two bits, increases on the rising edge of vs and in cycle {00, 01, 10, 11}. With different combinations of vs signals (vs_d, vs_i) and data signal (data_d, data_i), vs_o and data_o are output to the next module. These circuits allow for determining the detection area as shown in Figure 3.

The HOG feature values are calculated after regeneration of the synchronization signal and image data, being equal to those of the existing hardware oriented algorithm. HOG feature calculation comprises several sub-modules, namely, a three-line buffer, gradient calculation, 4× up-clocking, cell-based HOG feature calculation, block-based HOG feature accumulation, 4× down-clocking, normalization, and SVM classification modules. The main algorithm runs at 80 MHz, except for the cell-based HOG feature calculation and block-based HOG feature accumulation modules, which work at 320 MHz.

Before HOG feature calculation, surrounding pixels are cached in the three-line buffer module for gradient calculation. The output of this buffer comprises the surrounding pixels, and the calculation latency is 12-clock cycles. Gradient information calculation is conducted upon the output of the previous module, a 3-line × 66-pixel × 12-bit signal. Through 4× up-clocking, pixel parallelism reduces from 64 to 16 pixels per clock cycle. Then, the HOG feature values are calculated in each cell based on the obtained gradient magnitudes and orientations. Next, accumulation operations are performed in blocks, forming an 81-dimensional, vector, and sum of squares for each feature dimension is also accumulated. Normalization is conducted upon the 81-dimensional, descriptor and its summation value to increase robustness, in a process that takes 23 clock cycles. SVM classification is the last sub-module for HOG feature calculation upon blocks, and 3-clock cycles are required to obtain the final 32-bit HOG feature value. The adopted processing approach is detailed in [16].

**Figure 8:**
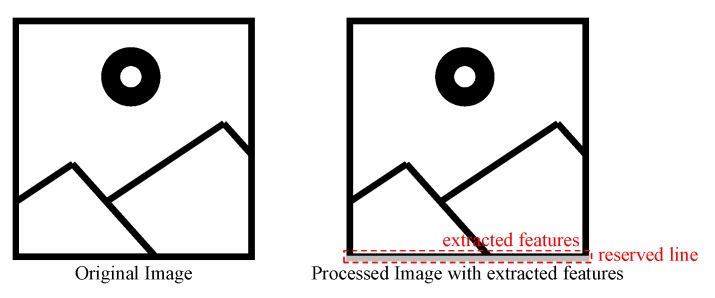
Original and processed images.

To improve efficiency, only the largest HOG feature value among the detection windows and its corresponding coordinates are recorded as shown in Figure 8. That is, the proposed method is more suitable for single object detection. In addition, the information of the four adjacent frames is stored. The location accuracy of detected objects can be improved through fusion of inter frame information. As the storage of HOG feature substantially reduces in the proposed algorithm compared to the original one, the additional information line is adequate to store the HOG feature value. The HOG feature value is represented with 32 bits, and the coordinates of the detected object are represented with 11 bits. The bit length is extended to 12 multiples for more efficient computing on the PC, matching the length of the image pixels. The HOG feature is a signed value, whose most significant bit is expanded, whereas coordinates require addition of a zero before the most significant bit to form a 12-bit value.

**Figure 9:**
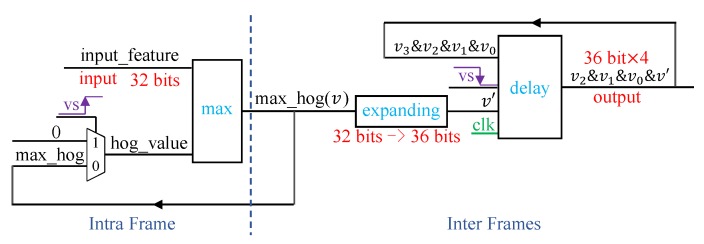
HOG feature storage for object detection.

Figure 9 shows the circuit diagrams for obtaining HOG feature values. This process can be divided into two parts, namely intra- and inter-frame processing. The input for intra-frame processing is the 32-bit HOG feature value of each detection window, and the maximum value is retrieved from comparison with previous ones. To ensure correctness, the maximum HOG feature value is set to zero at the beginning of the frame.

The maximum HOG feature value is expanded from 32 to 36 bits fulfilling three pixels to reduce the computation on the PC. At the end of the image frame indicated by the falling edge of signal vs, the 36-bit × 4 HOG feature values of four adjacent frames are output after applying shift and concatenation operations. The process is similar for coordinate values, and hence we omit its description.

## 5. Evaluation

To validate the proposed algorithm and its implementation, several evaluations are conducted upon our proposed algorithm. To put an emphasis on the novelty of our contribution, salience analysis and resource consumption are described in detail this section.

### 5.1. Salience Analysis

To determine whether objects located in the center of detection windows notably impact the results, we conducted a salience analysis representing the probability of objects contained in the detection window. As the highest salience is obtained when an object is located at the center of the detection window, we considered this situation as reference, 100% The salience of other object locations is defined as the proportion of the corresponding HOG feature value to the feature value from the centered object. Images of 48 × 48 pixels were cut around the center of the object with offset strides from 1 to 8 pixels. Including the image with centered objects in the detection window, we evaluated 289 images.

**Figure 10:**
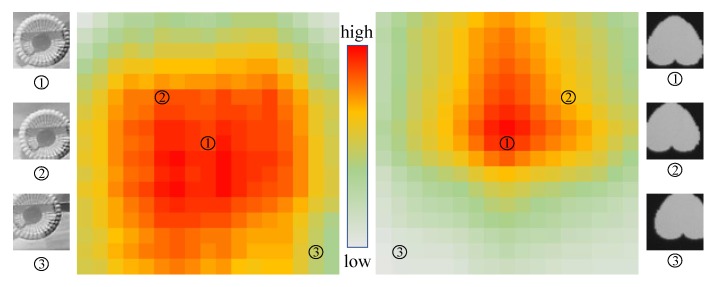
Salience trend as objects move away from the center of detection windows.

Two objects were selected for this evaluation, namely drogues and heart pattern (from a deck of cards). As expected, the results in Figure 10 show that salience reduces as the objects move away from the center of the detection window. Hence, objects are easily detected when located close to the center of the detection window. Some representative surrounding samples are also shown in the figure.

### 5.2. Resource Consumption

The Stratix FPGA chip used in this study contains 13,608 Kbits Block Memories (936 M9K and 36 M144K) and 832 MULT 18 × 18 Elements. The amount of some types of hardware resources, including LUTs, Register, Block Memories, MULT 18 ×18 Elements, are listed as shown in Table 1.

In this evaluation, hardware resource consumption focuses on Block Memories, where most of the M9K and M144K blocks are used. The surplus of distributed memories and dedicated multipliers can enable the proposed for algorithm expansion. Regarding resource consumption, the proposed algorithm is basically the same as the existing hardware oriented one. The detailed hardware resource consumption is listed in Table 2. There is a slight increment in resources of four-input LUTs and Registers compared with that in [16], and it is basically the same for resource of block Memories and MULT18×18.

### 5.3. Performance Evaluation

In this subsection, three different aspects of the proposed algorithms are evaluated, including detection accuracy, data throughput and execution time.

Accuracy is one of the most important indicators for object detection algorithms evaluation. We have evaluated the accuracy upon our self-built drogues database, which is collected in natural scenes. The evaluation is conducted with the assumption that the objects are located in the center of the images. Through training with 4500 images, including 1500 positive samples and 3000 negative samples, 999 of 1000 testing samples in total are classified correctly and the classification accuracy is 99.9%.

Data throughput and execution time are two other common indicators for high-speed vision systems evaluation. Ten-thousand images with size of 512 × 512 pixels are processed with FPGA in real-time. That is, the data throughput of the proposed system is about 29.3 Gbits and the execution time for each frame is 0.1 ms.

## 6. Experiments

**Figure 11:**
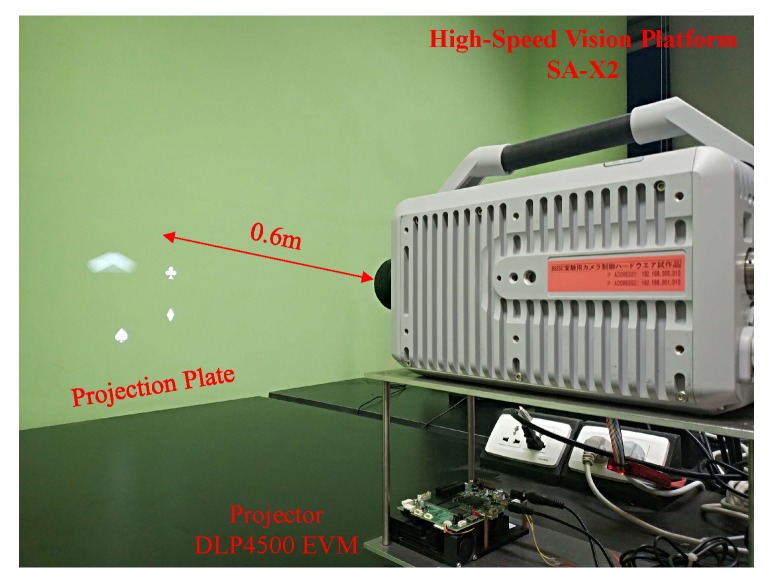
Overview of the experimental platform.

To verify the effectiveness of the proposed algorithm based on the HOG descriptor, we conducted several object detection experiments considering real scenarios. To prevent the difficult setup of using an ultra fast moving object, we generated the images using a high-speed projector (DLP4500 EVM; Texas Instruments, Dallas, TX, USA). The maximum frame rate of the projector is 4225 fps under binary mode, and the projector allows for synchronous trigger camera recording. To ensure adequate brightness, a frame rate of 2000 fps was selected in this evaluation, whereas the corresponding shutter speed was 1/2008 s, and a 50 mm f/1.8D optical lens (Nikon, Tokyo, Japan) was used for clear imaging. The experiment platform is shown in Figure 11. The projector was located directly below the camera, and the distance from the projection plane to the camera lens was approximately 0.6 m.

**Figure 12:**
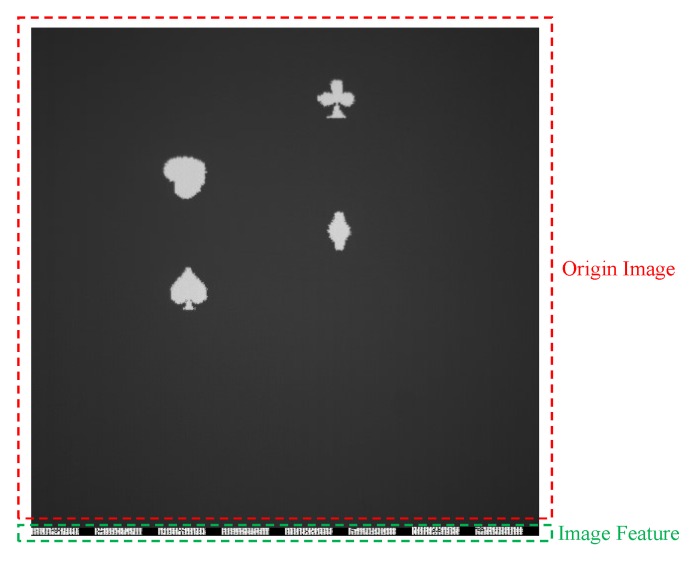
Sample of the captured frame.

In this evaluation, four different patterns (spade, club, diamond and heart from a deck of cards) were displayed on the plane with the projector, and our goal was to detect the heart pattern. The size of the patterns is about 48 × 48 pixels in the captured images, being equal to the size of the detection windows. Figure 12 shows a sample of original captured image, where the calculated features are stored in the bottom of the images.

The heart pattern was moved regularly at different speeds. First, the heart pattern moved from the upper-left to the lower-right, then horizontally to the left, and finally it moved upwards obliquely to reach the starting point. The other three image patterns remained fixed in the scene.

**Figure 13:**
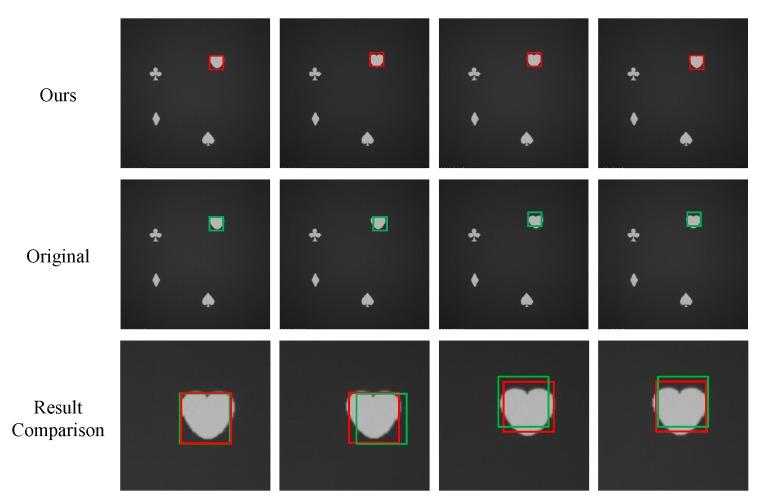
Detected location of an object retrieved from proposed and existing algorithms.

Figure 13 shows the detection results of the proposed algorithm and the existing one in an experiment. The first row corresponds to the detection results of the proposed algorithm, with the objects being framed in red rectangles. The results of the existing algorithm are displayed in the second row, with the detection windows being shown as green rectangles. For a better comparison of the accuracy between the proposed algorithm and the existing one, a small area around the heart pattern is selected, and the detection results of both algorithms are displayed in the same picture at the last row.

Two experiments as displayed in Video S1 were conducted to account for objects moving at low and high speeds. The coordinate values are the results of adjacent-frame information fusion for the first experiment, while, for the second experiment, the coordinate values of the object in the current frame are the final results. One thousand consecutive frames were stored to determine the detection accuracy of the proposed algorithm.

In the first experiment, the object moved at a relatively low speed, with the object displacement between consecutive frames being below three pixels.

**Figure 14:**
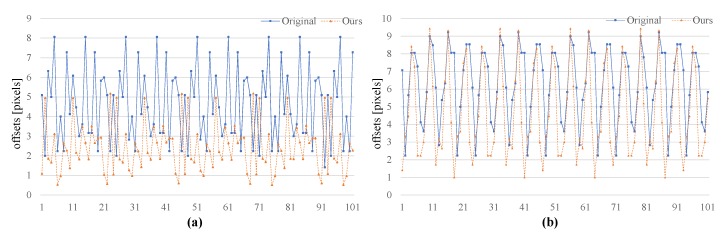
Difference between detected and real location of object in the two evaluated algorithms. (**a**) at low object speed; (**b**) at high object speed.

**Figure 15:**
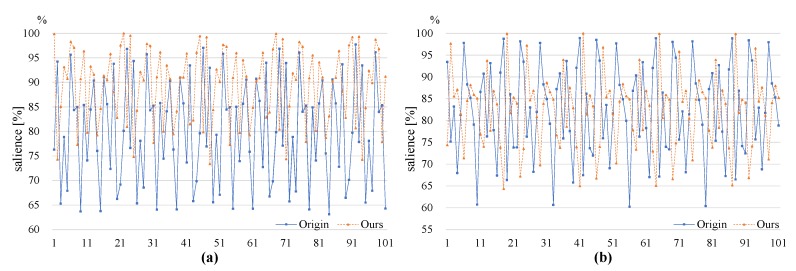
Salience comparison between the two evaluated algorithms. (**a**) at low object speed; (**b**) at high object speed.

**Figure 16:**
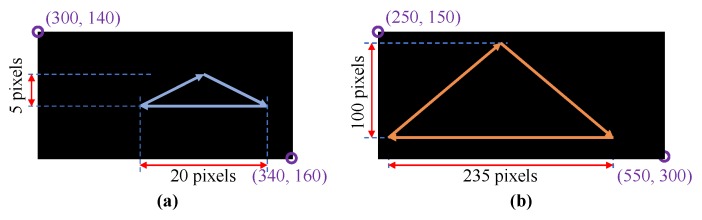
(**a**) moving trail of the first experiment; (**b**) moving trail of the second experiment.

The average difference between the detected and real location of the object is 4.62 pixels using the original algorithm and 2.37 pixels using the proposed algorithm. In the second experiment, the object moved at a relatively high speed, with displacements above eight pixels between consecutive frames. In this case, the average difference between the detected and real locations of the object is 5.84 pixels using the original algorithm and 5.64 pixels using the proposed method. Hence, the detection accuracy of the proposed algorithm improves notably for objects moving at low speed, as their position in previous frames can be used for multiple-frame compensation. Figure 14 shows the difference between detected and real location of the first 100 frames in the two evaluated algorithms at low and high speeds. The comparison of salience in each detection window using the two algorithms at the two speeds is shown in Figure 15. Finally, the moving trails in these two experiments are presented in Figure 16. The blue and orange lines represent the results from the original and proposed algorithms, respectively.

**Figure 17:**
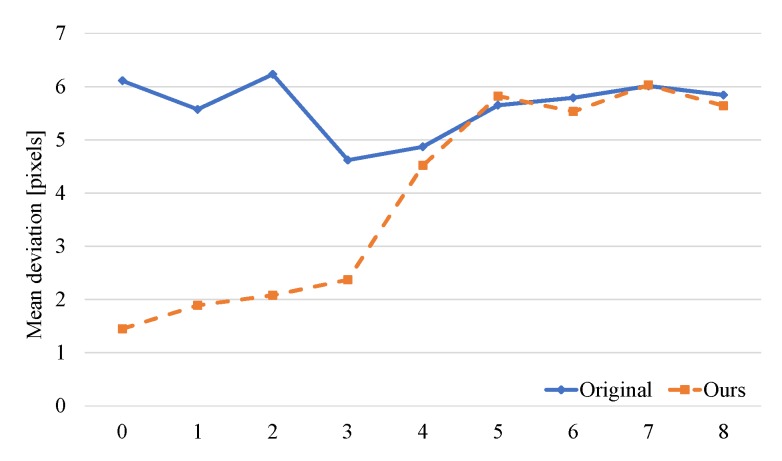
Mean deviations for different object displacements between frames.

In order to further illustrate the effectiveness of the proposed method, there are several experiments in which object displacements between consecutive frames are in a range of 0 to 8 pixels. The mean deviation between detected coordinates and true values are calculated and displayed as in Figure 17. The deviations shown with the blue line are the result of the existing algorithm and that in orange is the result of the proposed method. Through multi-frame information fusion, our proposed method performs better when displacements between consecutive frames are less than 3 pixels. In other words, the proposed algorithm is highly accurate at a relatively low object speed and can maintain a high accuracy when the objects moves faster.

## 7. Conclusions

Facing the remaining problem that large strides exist between detection windows, a multi-frame information fusion method is proposed in this paper. Compared with the existing algorithm, the localization deviation of the proposed method is reduced based on the detection information of adjacent frames. For improved processing efficiency, only the maximum HOG feature and corresponding coordinate values in the frame will be stored in the bottom of images together with adjacent frames. Several experiments have demonstrated that our proposed method has an effect when the moving speed of the object is relatively low and the accuracy is maintained when it moves faster.

Nevertheless, the proposed design is only applicable to single-scale object detection and lacks scale invariance. In the future development, we will expand the algorithm to a multi-scale one for improving robustness against object scales changing.

## Figures and Tables

**Table 1:** Resource consumption of all modules.

Device Type	Ours	[16]	Total Resource	Percent (Ours)
4-input LUTs ${}^{*}$	320,665	313,349	524,160	61.18%
Registers	96,822	89,260	232,960	41.56%
M9K blocks	860	859	936	91.88%
M144K blocks	36	36	36	100.00%
Embedded Mems (kbit)	9593	9637	20,726	46.28%
MULT18×18	268	268	832	32.21%

* Assume that ALM equals to two 4-input LUTs.

**Table 2:** Resource usage of each submodule in the digital image processing module.

Module	4-input LUTs ${}^{*}$	Register	Memory Bits	Multipliers
vs-signal regeneration	3183	2638	0	0
Lines Buffer	7338	5920	0	0
Gradient Calculation	127,762	26,556	0	128
Up-Clocking	983	574	8064	0
Cell-based HOG Feature	14,534	5568	0	0
Block-based HOG feature	31,067	21,353	0	0
Normalization	77,583	11,899	39,590	0
SVM Classification	4126	2236	0	108
**Total**	**266,023**	**77,845**	**47,654**	**236**

* Assume that ALM equals to two 4-input LUTs.

## Supplementary Materials

The following are available online at https://www.mdpi.com/1424-8220/19/17/3707/s1, Video S1: Multi-frame information fusion based ultra-high-speed object detection.
